# Rare presentation of multicentric sebaceous carcinoma of the breast in a 33-year-old woman: diagnostic challenges and treatment approach

**DOI:** 10.3332/ecancer.2025.1908

**Published:** 2025-05-15

**Authors:** Izaberen Sampaio Estevam, Mariana Macambira Noronha, Júlia Matos Dubanhevitz, Eric Lima Freitas Mota, Ígor Giordan Duarte Jorge, Conceição Aparecida Dornelas, Diane Isabelle Magno Cavalcante, Georgia Fiuza Alencar Araripe, Márcio Marcondes Vieira

**Affiliations:** 1 Universidade Federal do Ceará, Fortaleza 60430-160, Brazil; 2 Centro Regional Integrado de Oncologia, Fortaleza 60335-480, Brazil; 3 Grupo de Educação e Estudos Oncológicos, Fortaleza 60430-230, Brazil; a https://orcid.org/0009-0003-6292-2393; b https://orcid.org/0000-0003-0889-7425; c https://orcid.org/0009-0001-3708-3546; d https://orcid.org/0009-0004-7238-9710; e https://orcid.org/0000-0002-5237-1271; f https://orcid.org/0000-0002-1692-086X; g https://orcid.org/0000-0001-7585-0307

**Keywords:** adenocarcinoma sebaceous, triple negative breast neoplasms, case reports

## Abstract

Background: Sebaceous carcinoma of the breast (SCB) is an unusual neoplasm. To the best of our knowledge, only 30 cases have been reported in the literature. Due to its rarity, there is limited knowledge on how to manage patients with SCB. This article aims to describe a case of multicentric sebaceous breast carcinoma in a 33-year-old woman detailing the diagnostic process, historical findings and the treatment approach.

## Introduction

Sebaceous carcinoma (SC) is a rare type of cutaneous tumour originating in the sebaceous glands and occurring more frequently after age 60 [1]. Tumours of sebaceous origin are usually found on the eyelid, head and neck, less than 30 cases have been described of breast origin [2, 3]. Due to its low incidence, the behavior and prognosis of this special type of breast cancer are still poorly understood. This is a case report of a 33-year-old representing the second reported case of this tumour in a patient under 40 years of age [4].

## Case report

In December 2019, a 33-year-old Brazilian woman, with no family history of cancer, presented to a Mastology Department in Ceará, Brazil, having palpated a lump in her right breast 2 months prior. Breast ultrasound demonstrated a solid, irregular, hypoechoic and lobulated nodule in the upper lateral quadrant, measuring 2.4 × 1.4 cm (BI-RADAS 4).

**Figure 1.**
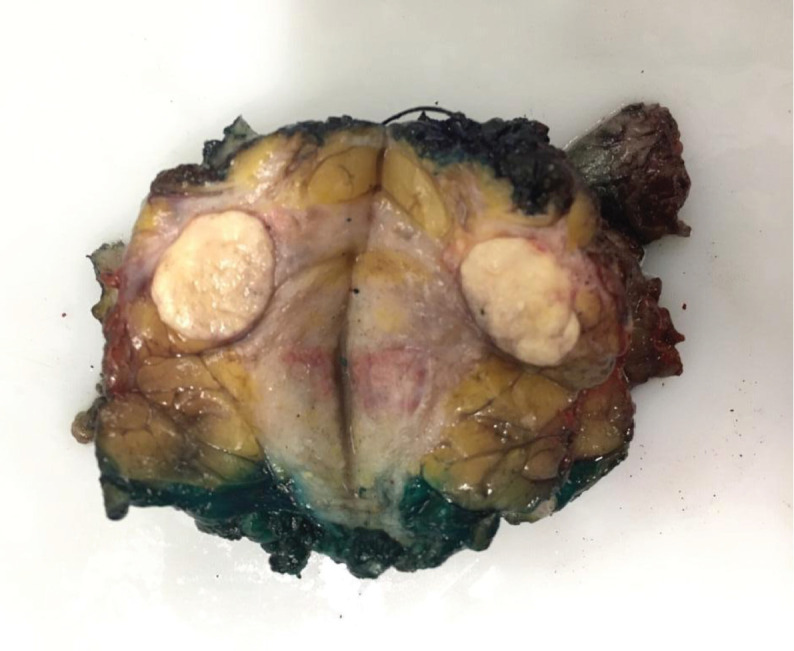
Breast specimen of the tumour demonstrating a multicentric manifestation.

**Figure 2.**
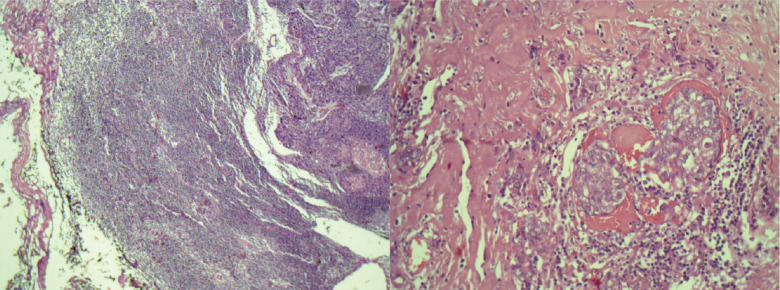
Histologic section of the tumour in the breast, demonstrating a sebaceous differentiation.

**Figure 3.**
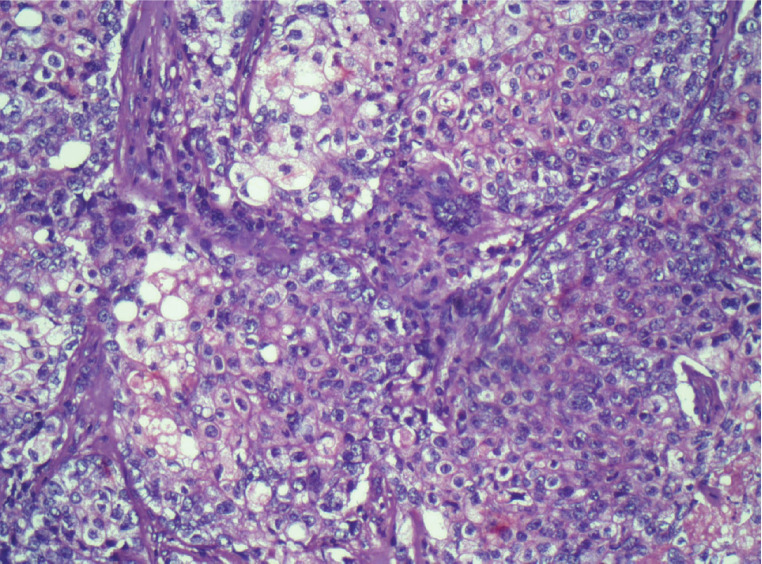
Histologic section of the lymph node metastasis, demonstrating a sebaceous differentiation.

Another solid, oval nodule parallel to the skin, in the 4 o’clock radius, measuring 1 × 0.7 cm. Axillary ultrasound presented lymph node enlargement in the right axilla measuring 2.5 × 1 cm. A core biopsy of the largest nodule was performed, and the result indicated a Nottingham grade 3 invasive carcinoma, with areas of sebaceous differentiation. Immunohistochemistry showed negativity for estrogen receptor, progesterone and human epidermal growth factor (HER2) and positivity for epithelial membrane antigen (EMA), with Ki67 of 50%, representing SC with a triple-negative phenotype. The patient underwent quadrantectomy with sentinel lymph node biopsy. During surgical manipulation, another nodule, not previously described in imaging studies, was found approximately in the 12 o’clock radius of the same breast, which was also removed (Figure 1). The anatomopathological study confirmed the diagnosis of sebaceous carcinoma of the breast (SCB) (Figure 2) – grade 3, with the superficial margin compromised by the neoplasia and metastasis of carcinoma in 1 axillary lymph node (Figure 3). The other nodule corresponded to invasive carcinoma of non-special subtype grade 2, with a high-grade *in situ* component and narrow surgical margins, characterising it as a multicentric disease. The immunohistochemical profile of the removed nodule confirmed the triple-negative phenotype of the tumour, with Ki67 of 60% and histopathological description consistent with SCB as well. Given the diagnosis of multicentric disease, the patient underwent nipple-sparing mastectomy in the right breast, with axillary dissection and reconstruction with a prosthesis, performed 1 month after the first surgery. The anatomopathological study of the specimen demonstrated free margins and confirmed residual SC in the surgical specimen. The patient proceeded with adjuvant chemotherapy, consisting of weekly paclitaxel for 12 weeks, followed by dose-dense doxorubicin and cyclophosphamide administered every 21 days for 4 cycles and adjuvant radiotherapy to the right breast, having received a dose of 50.4 Gy in 28 fractions with 6 MV. A germline test was requested to evaluate hereditary predisposition to breast and ovarian cancer, without evidencing pathogenic or probably pathogenic variants. The patient underwent follow-up for 5 years, including semi-annual thoracic and abdominal computed tomography scans and mammography, with no evidence of neoplastic recurrence.

## Discussion

Sebaceous breast carcinoma (SBC) is an extremely rare occurrence, even more in young patients [4]. Until the 4th edition of the World Health Organisation (WHO) tumour classification editorial in 2012, SBC was classified as an exceptionally rare special type of breast tumour, defined as the presentation of sebaceous differentiation in at least 50% of the cells, with no evidence of skin or nipple origin [5]. As of 2019, the WHO classifies SC as a histological pattern of invasive carcinoma of no special subtype, due to insufficient clinical evidence to classify it as a special subtype of breast neoplasia [6].

The diagnosis of SBC is made based on histological and immunohistochemistry studies, and some differential diagnoses include lipid-rich carcinoma, glycogen-rich carcinoma, carcinoma with apocrine differentiation and metastatic clear cell renal carcinoma [7]. Notably, most SBCs exhibit positivity for estrogen and progesterone receptors or HER2, with a minority presenting as triple-negative, as observed in this case [8]. Interestingly, some studies indicate an 88% prevalence of EMA positivity in SBC, making it a crucial marker for this diagnosis [9].

Cuteanos SC can be linked to Muir–Torre syndrome (MTS), an autossomic and uncommon variant of Lynch syndrome, that is characterised by multiple sebaceous gland tumours and lifetime high risk for colorectal and endometrial cancer, as a result of pathogenic variants in the mismatch repair genes [10, 11]. While SBC can be associated with MTS or even pathogenic variants in *BRCA* genes, the young patient in this case did not have any known predisposition for hereditary cancer [12, 13].

As it is a rare tumour, there is little epidemiological data, but it is suggested that SCB should be considered a neoplasm with a high degree of malignancy, some cases present with an aggressive clinical course, including lymph node and distant metastases [4, 14]. Even though this patient had a triple-negative component, which is associated with a poor prognosis, early detection and appropriate treatment led to a positive outcome. A key limitation of this study was the inability to perform next-generation sequencing due to resource constraints within the Brazilian public healthcare system (SUS), precluding valuable molecular profiling of the tumour.

## Conclusion

This report highlights the rarity of SCB, especially in patients under 40 years of age. Due to its low incidence, the behavior and prognosis of this subtype are poorly understood, making detailed case descriptions essential, especially to guide the management of similar cases. In the case of the patient described, the management included surgical removal of the disease, which was multicentric, followed by adjuvant chemotherapy and radiotherapy. This study contributes valuable knowledge to the field, potentially guiding physicians in the diagnosis and management of similar cases in the future.

## List of abbreviations

EMA, Epithelial membrane antigen; HER2, Human epidermal growth factor; MTS, Muir-Torre syndrome; SC, Sebaceous carcinoma; SCB, Sebaceous carcinoma breast; WHO, World Health Organisation.

## Conflicts of interest and source of funding

All authors report no relationships that could be construed as conflicts of interest. All authors take responsibility for all aspects of the reliability and freedom from bias of the data presented and their discussed interpretation. There was no source of funding.

## Informed consent

Written consent for publication was obtained from the patient.
